# Supplementary material for: Effects of the Transfer Method and Interfacial Adhesion on the Frictional and Wear Resistance Properties of a Graphene-Coated Polymer
Source: Nanomaterials (Basel). 2023 Feb 8;13(4):655. doi: 10.3390/nano13040655 (PMC9967740; doi:10.3390/nano13040655)
Supplement: Supplementary file 1 [file nanomaterials-13-00655-s001.zip › nanomaterials-2138613-supplementary.pdf]

Supplementary Information

Effects of Transfer Method and Interfacial Adhesion on Frictional and Wear Resistance Properties of Graphene-Coated Polymer

Temesgen B. Yallew [1, 2, 3], Prashant Narute [1, 3], Rakesh S. Sharbidre [1, 3], Ji Cheol Byen [1, 4], Jaesung Park [1] and Seong-Gu Hong [1, 4, *]

[1] Interdisciplinary Materials Measurement Institute, Korea Research Institute of Standards and Science, Daejeon 34113, Republic of Korea.

[2] School of Mechanical and Industrial Engineering, Mekelle University, Mekelle 231, Ethiopia.

[3] Physiological Signal Processing and Measurement Solution, Physionics, Daejeon 34027, Republic of Korea.

[4] Department of Nano Science, University of Science and Technology, Daejeon 34113, Republic of Korea.

* Correspondence: sghong@kriss.re.kr; Tel.: +82-42-868-5868

Table S1. Friction coefficient of Gr/PET films and bare PET substrate as a function of scratch distance.

Scratch distance (mm)	Friction coefficient				
	DT (APS+IM)	DT (APS)	WT (APS+IM)	WT (APS)	Bare PET
0.1	0.13 (±0.01)*	0.15 (±0.02)	0.19 (±0.03)	0.28 (±0.05)	0.35 (±0.10)
0.2	0.28 (±0.03)	0.31 (±0.04)	0.33 (±0.08)	0.41 (±0.08)	0.46 (±0.10)
0.3	0.30 (±0.03)	0.39 (±0.04)	0.39 (±0.05)	0.46 (±0.06)	0.54 (±0.10)
0.4	0.36 (±0.03)	0.44 (±0.04)	0.44 (±0.05)	0.49 (±0.07)	0.57 (±0.07)
0.5	0.39 (±0.02)	0.45 (±0.03)	0.45 (±0.05)	0.51 (±0.05)	0.57 (±0.08)

* Average value (standard deviation)

Table S2. Penetration depth of Gr/PET films and bare PET substrate as a function of scratch distance.

Scratch distance (mm)	Penetration depth (nm)				
	DT (APS+IM)	DT (APS)	WT (APS+IM)	WT (APS)	Bare PET
0.1	352 (±22)*	373 (±21)	413 (±39)	476 (±71)	709 (±106)
0.2	610 (±25)	632 (±24)	746 (±68)	843 (±130)	1318 (±206)
0.3	828 (±32)	858 (±39)	1052 (±137)	1171 (±187)	1877 (±314)
0.4	1019 (±39)	1050 (±46)	1331 (±168)	1483 (±236)	2389 (±402)
0.5	1195 (±47)	1226 (±58)	1572 (±186)	1792 (±294)	2886 (±492)

* Average value (standard daviation)

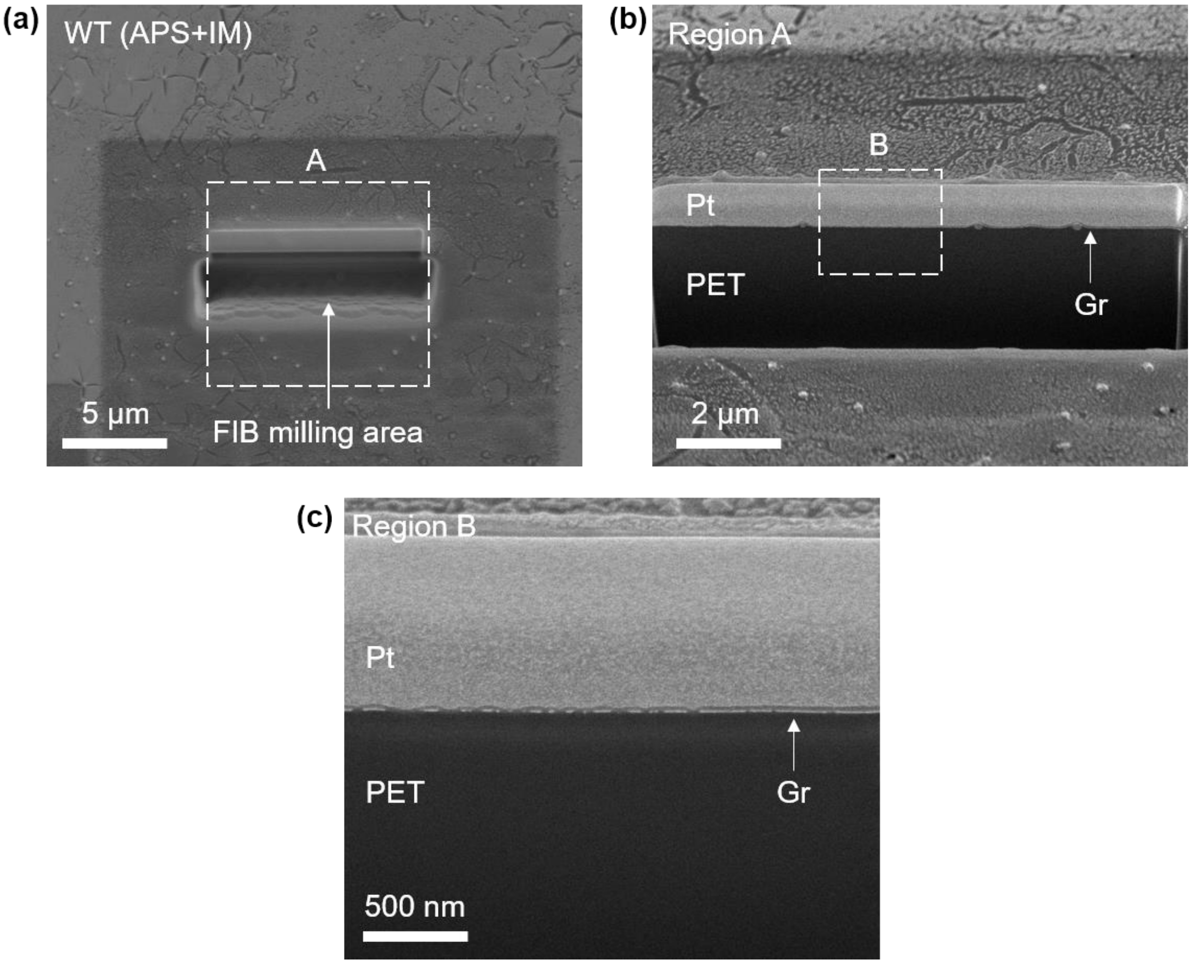

Figure S1. SEM micrographs showing the cross-section of WT (APS+IM) sample, where a 10-nm-thick Pt layer was deposited on the sample surface and then focused ion beam (FIB) milling was applied to cut the sample. (a) FIB milling area, (b) enlarged view of region A in (a), and (c) enlarged view of region B in (b).

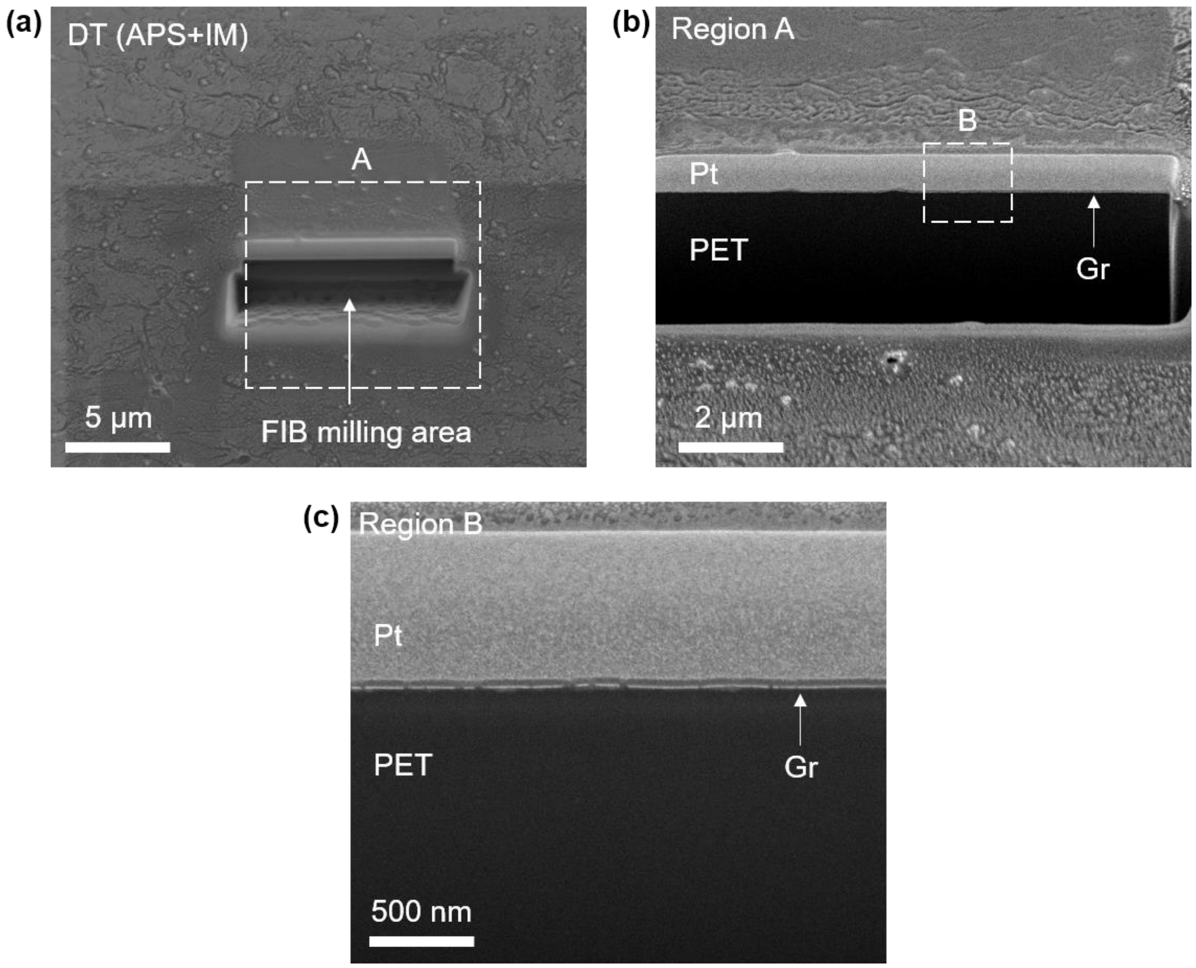

Figure S2. SEM micrographs showing the cross-section of DT (APS+IM) sample, where a 10-nm-thick Pt layer was deposited on the sample surface and then FIB milling was applied to cut the sample. (a) FIB milling area, (b) enlarged view of region A in (a), and (c) enlarged view of region B in (b).

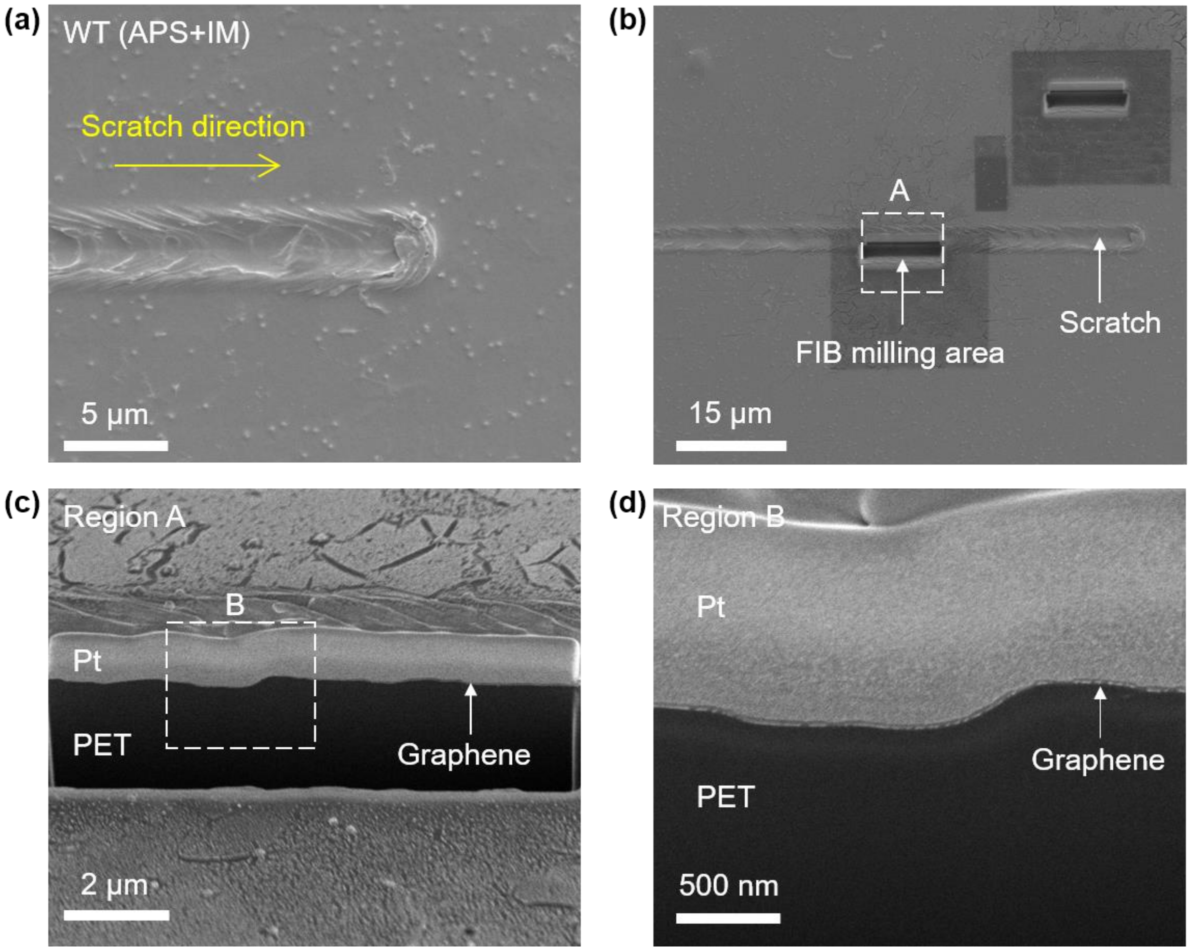

Figure S3. SEM micrographs showing the cross-section of the scratch on WT (APS+IM) sample, where a 10-nm-thick Pt layer was deposited on the sample surface and then FIB milling was applied to cut the sample. (a) 50° tilted image of the end part of the scratch after Pt deposition, (b) FIB milling area on the scratch, (c) enlarged view of region A in (b), and (d) enlarged view of region B in (c).

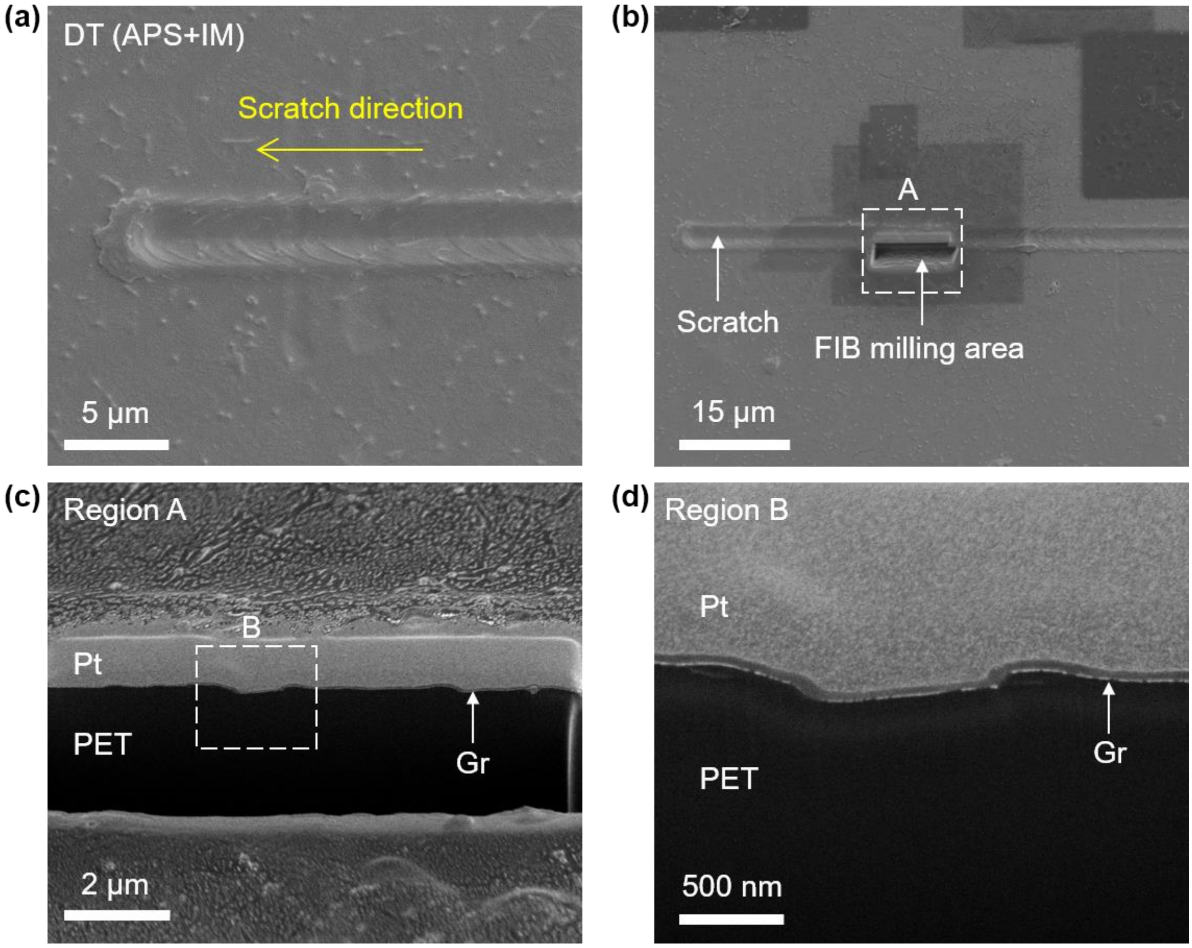

Figure S4. SEM micrographs showing the cross-section of the scratch on DT (APS+IM) sample, where a 10-nm-thick Pt layer was deposited on the sample surface and then FIB milling was applied to cut the sample. (a) 50° tilted image of the end part of the scratch after Pt deposition, (b) FIB milling area on the scratch, (c) enlarged view of region A in (b), and (d) enlarged view of region B in (c).

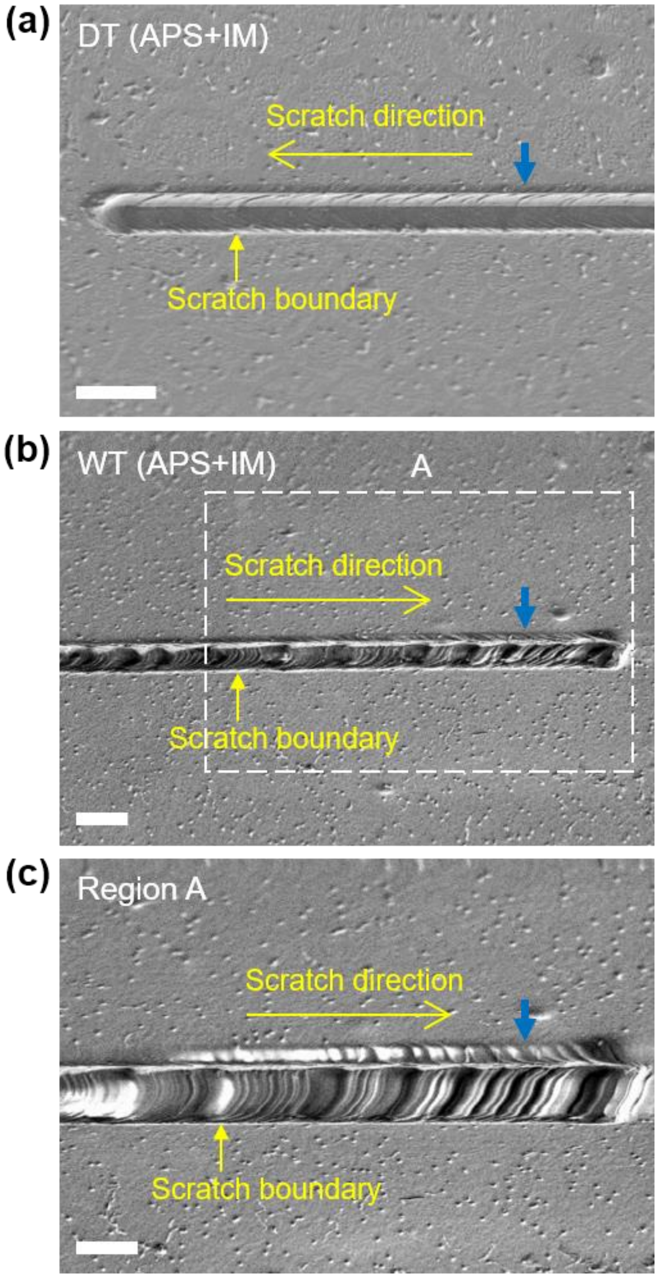

Figure S5. 45° tilted SEM micrographs showing the scratch traces of (a) DT (APS+IM) and (b) WT (APS+IM) samples. (c) Enlarged view of region A in (b). Scale bars: 5 μm. Blue arrows indicate the boundary region of the scratch where a more pronounced delamination feature of Gr layer is observed in WT (APS+IM) sample than DT (APS+IM) sample.

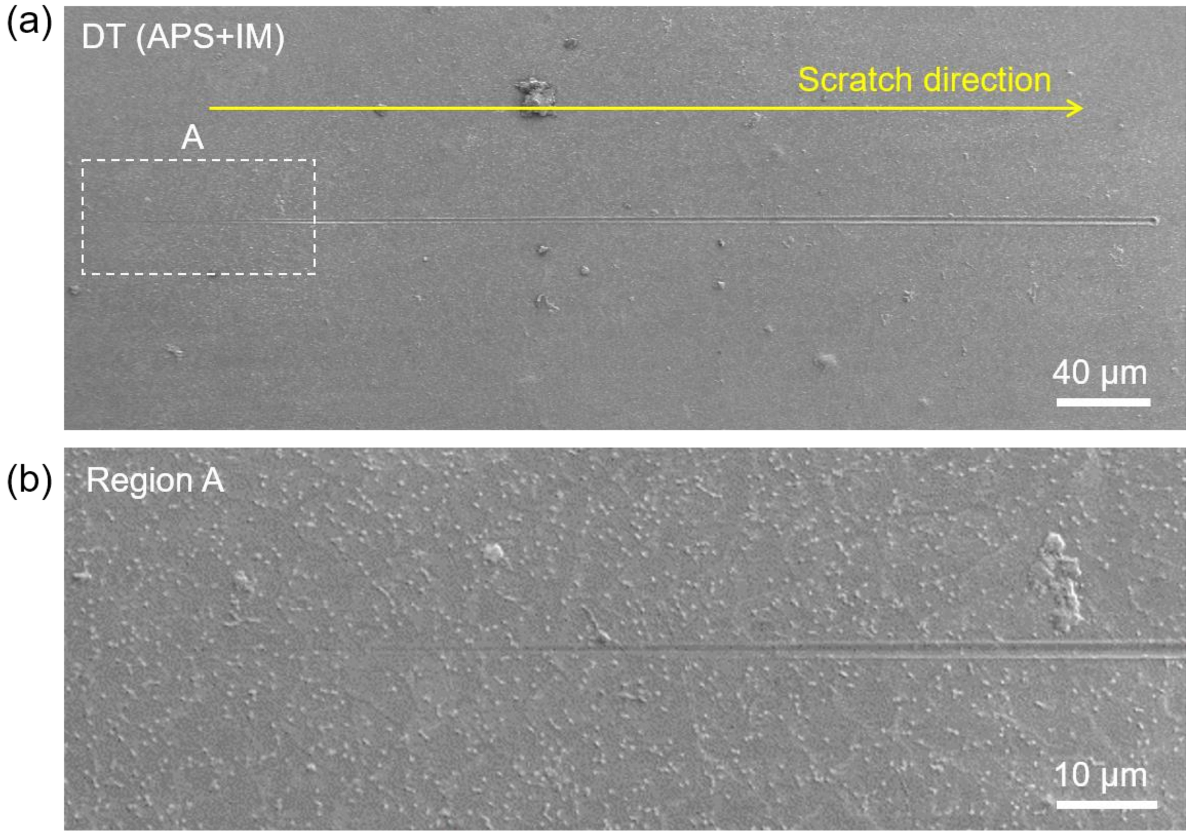

Figure S6. SEM micrographs showing the scratch trace of DT (APS+IM) sample. (a) Overall scratch trace and (b) enlarged view of the initial stage of the scratch (region A in (a)).
